# New formulation of ibuprofen on absorption-rate: A comparative bioavailability study in healthy volunteers

**DOI:** 10.22088/cjim.10.2.150

**Published:** 2019

**Authors:** Yasaman Moghadamnia, Sohrab Kazemi, Boshra Rezaee, Mehrdad Rafati-Rahimzadeh, Soheil Ebrahimpour, Fahimeh Aghapour

**Affiliations:** 1 Alzahra University, Tehran, Iran; 2 Cellular and Molecular Biology Research Center, Health Research Institute, Babol University of Medical Sciences, Babol, Iran; 3 Mobility Impairment Research Center, Health Research Institute, Babol University of Medical Sciences, Babol, Iran; 4 Infectious Diseases and Tropical Medicine Research Center, Health Research Institute, Babol University of Medical Sciences, Babol, Iran; 5 Neuroscience Research Center, Health Research Institute, Babol University of Medical Sciences, Babol, Iran

**Keywords:** Ibuprofen, Pharmacokinetics, COX2-inhibitory effects

## Abstract

**Background:**

Enteric-coated capsules are solid dosage forms which are designed to bypass the stomach and release the drug in the small intestine. This study was done to compare pharmacokinetics of ibuprofen tablet and ibuprofen as enteric-coated capsule using sodium alginate beads.

**Methods:**

A crossover randomized study was performed on 12 healthy volunteers receiving a single dose of regular ibuprofen tablet (200 mg) and enteric-coated capsule (200 mg). The washout time between the periods was one month. Pharmacokinetic and pharmacodynamic blood samples were collected for 16 hours following treatment. High-performance liquid chromatography (HPLC) method used the following specifications: C18 column with 4.6 mm diameter & 25 mm length, the fluorescent detector of excitation and emission wavelengths were 224 and 290 nm, respectively.

**Results:**

After a single oral dose of ibuprofen formulations, the median times to maximum concentration were 60 and 240 minutes in ibuprofen tablet (200 mg) and enteric-coated capsule, respectively. The maximum levels for the participants receiving ibuprofen tablet and enteric-coated capsule were 11.71±1.3 and 10.32±4.19 µg/mL, respectively. The pharmacokinetic (PK) modeling data showed the area under curve (AUC) to be 61.51 hours & 86.62 hours for the group receiving the tablet and the capsule, respectively.

**Conclusion:**

According to the results, in is concluded that enteric coating may delay the onset of ibuprofen effect and increases the duration of action. This formulation has advantages over the conventional drug delivery systems as it lengthens the dosing intervals and also increases patient compliance for chronic pain.

Ibuprofen is a widely known member of non-steroidal anti-inflammatory drugs (NSAIDs). It is mostly used as a relieving agent in acute and chronic pains and inflammation. Common adverse effects of the NSAIDs e.g. ibuprofen may include gastrointestinal (GI) symptoms, such as stomachache, heartburn, gastric ulcers and bleeding (1-3). These effects mostly occur because ibuprofen is a none-specific COX inhibitor (4-6). Following oral administration, ibuprofen tablet is disintegrated and dissolved in the gastric fluids, which may lead to such painful side effects (7, 8). Most analgesics are formulated to reach higher blood concentration in a lower duration, to relieve the acute or sub-acute pain more rapidly (9, 10). However, in the case of patients with chronic inflammatory conditions that experience mild to moderate pain, achieving a formulation that can maintain steady- state concentrations in blood, is more effective in treatment (11, 12).

To overcome the above mentioned problems, it is suggested to encapsulate ibuprofen in sodium alginate beads, coated with calcium chloride (13, 14). Such a formulation will help to bypass disintegration in gastric fluids, directly reaching to the alkaline medium of duodenum. In addition to avoiding the gastrointestinal pains caused by the conventional formulation, this method will also help maintain a reasonable blood concentration and therefore have more long-term effects. However, due to the formulation of ibuprofen and the pharmacokinetics of different formulations, the effective treatment with COX2 inhibition has been established previously (15). This clinical study was done firstly to investigate a special formulation for designing sodium alginate that can thoroughly encapsulate ibuprofen formulations evaluated at a dose of 200 mg, the usual prescribed dose for an analgesic effect, and the pharmacodynamic effects were compared based on time–COX inhibition relationships. 

## Methods

**Study population and design: **This clinical trial was a randomized, single-dose, open-label, tow-treatment study performed on 12 healthy medical student volunteers (age 19–20 years). Participants were listed according to the following condition: weight >50 kg, body mass index of 18.5–27 kg/m^2^, Absence of clinically considerable medical history, physical examination detection, 12-lead electrocardiogram readership, or clinical laboratory-testing consequences, containing serum chemistry, hematology, urinalysis, and infected serology. The contexts and purpose were entirely demonstrated, and written informed consent was obtained. This study was approved by the Institutional Review Board (IRB) and Medical Ethics Committee of Babol University of Medical Sciences, which was carried out according to the Proclamation of Helsinki Good Clinical Practice.

**Chemicals and standards** **: **All chemicals and solvents were of analytical grade which was purchased from Merck, Germany and the high-performance liquid chromatography (HPLC) grade solvents that were purchased from Dae-Jung, Korea. Ibuprofen (batch number 450025) was a gift from the Caspian Tamin Pharmaceutical Company (Iran).

**Preparation of chitosan-alginate as a base bead** **: ** Sodium alginate and calcium chloride solutions were prepared to be dissolved in distilled water. The pH of the sodium alginate solution was adjusted at 5.1 using 0.1 N hydrochloric acid. Chitosan was dissolved in 1% acetic acid solution, and the pH was modified at 5.4 using 0.1 N NaOH. Two milliliters of calcium chloride solution (3.35 mg/ml) was added gently to 10 ml aqueous sodium alginate solution (3.0 mg/ml) and stirred for 30 min, and then 4 ml chitosan solution (3.0 mg/ml) was added to the final calcium alginate gel and stirred for an additional 1 hour (16, 17). 

**Figure 1:**
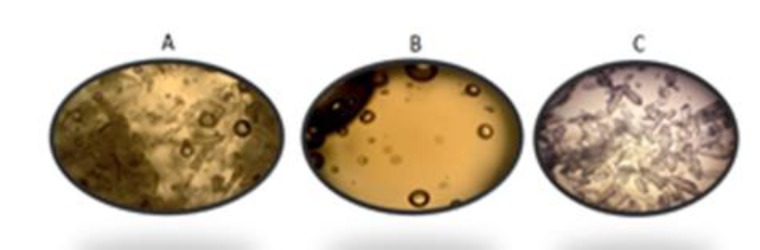
Microscopic image of A) ibuprofen loaded beads, B) beads without ibuprofen and C) ibuprofen powder

**Preparation of ibuprofen loaded chitosan-alginate** **beads: **A volume of 300 µl ibuprofen solution (1.105 mg/ml of an equal proportion of ethanol and water mixture), was incorporated into the calcium chloride solution. The remaining process was performed similarly to the preparation of chitosan-alginate beads without ibuprofen (figure 1). 

**HPLC system and operating conditions** **: **High performance liquid chromatography (HPLC, Knauer, Germany) equipped with the fluorescent detector (Shimadzu, Japan) was used to measure the concentration of ibuprofen in serum samples. The working conditions were as follows: the excitation and emission wavelengths were 224 and 290 nm, respectively. The column was C18 (Eurospher), and the mobile phase was a mixture of acetonitrile and water in a ratio of 40/60 acetonitrile and aqueous phosphoric acid 0.2 (v/v %). The temperature was 25 °C, and the flow rate of the mobile phase was 1 ml/min. Ezchrome software was used to extract area and height of the peaks. The slope, intercept, R^2 ^coefficient and the coefficient variation (CV) were calculated for this analysis (18).

**Disintegration testing in phosphate buffers: **For recognizing the buffer molarities corresponding to physiological bicarbonate buffers, disintegration test was used as a rapid screening test. The test was carried out using a hotplate stirrer and HPLC, for doing this experiment, first, 10 cc of the buffer solution with PH 7.4 was transferred to an erlenmeyer 50 cc, and then 0.1 g of synthetic ibuprofen was added to the buffer. 100 μl of the supernatant solution was taken, and 20 μl of it was injected into the chromatography. This experiment was repeated in 0.5, 1, 2, 4 and 8-hour time and then, the experiment was carried out in a buffer with PH 2 that was similar to the gastric acidic environment. Then, in 0.5, 1, 2, 4 and 8 hours, the ibuprofen diffusion rate was measured by chromatography, and the results of the chromatographic analysis were calculated. 

**Pharmacokinetic and pharmacodynamics assessment: **For the pharmacokinetic evaluation the blood samples were obtained before dosing (0 hours) and 0.5 ,1, 1,2 ,4 ,8 ,12, and 16 hours after administration of the drug in each term. At these, 6 mL of blood was extracted. The blood was poured into a sodium heparin tube and centrifuged at 3,000 rpm at 4°C for 10 minutes to evaluate pharmacokinetics. To analyze separated plasma samples, they were frozen and stored at -70°C. Using high-performance liquid chromatography, pharmacokinetic samples were analyzed. Plasma concentrations of ibuprofen were measured by a sensitive, specific and validated HPLC method using fluorescent detector. After thawing, each sample solution was prepared in acetonitrile and aqueous phosphoric acid 0.2 (v/v %). The organic layer decanted and evaporated under the nitrogen stream at 40^o^C. The dried extract was then reconstituted with 0.5 ml of acetonitrile and aqueous phosphoric acid 0.2 (v/v %) 60:40 (v/v) mixture.

**Pharmacokinetic and pharmacodynamic analysis: **Using the noncompartmental analysis of WinNonlin® 6.4 pharmacokinetic parameters for ibuprofen were calculated (Certara, Princeton, NJ, USA). The plasma concentration–time profiles were used to obtain maximum observed plasma concentration (Cmax) and the time to the maximum concentration was observed in plasma (Tmax). After the administration of the drug, the area under the plasma concentration–time curve was estimated by the linear-up and log-down trapezoidal method. The slope of the terminal log-linear phase was the elimination rate constant (λz), and by ln (2)/λz the terminal elimination half-life (t½) was calculated (19). 

**Figure 2:**
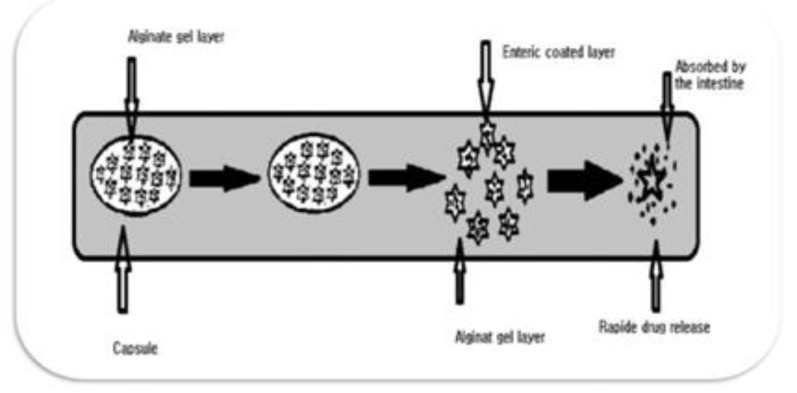
Design of enteric-coated timed-release Capsule

**Mechanism of the enteric-coated time-release capsule** **: **Capsule is composed of three layers, a drug containing core capsule (rapid release function), the press coated polymer layer (Chitosan-Alginate beads layer, time release function) and an enteric coating layer (acid resistance function). The enteric coating layer does not release the drug in the stomach due to the acid resistance of the outer enteric coating layer. The enteric coating layer rapidly dissolves after gastric emptying and the intestinal fluid begins to slowly erode the press coated polymer layer. Rapid drug release occurs when the erosion front reaches the core polymer layer since the erosion process takes a long time as there is no drug release period (lag phase) after gastric emptying. The period of drug release period is controlled either by the weight or composition of the polymer layer (figure 2).

**Statistical Analysis: **This analysis was performed using SPSS software Version 16. Statistical analyses were performed using one-way ANOVA followed by post-hoc Tukey test. To compare the geometric mean ratios of AUC0–t and Cmax pharmacokinetic values with the 90% confidence intervals (CIs), analysis of variance was performed using a mixed-effect model. The level of significance P<0.05 was considered statistically significant.

## Results

**Figure 3:**
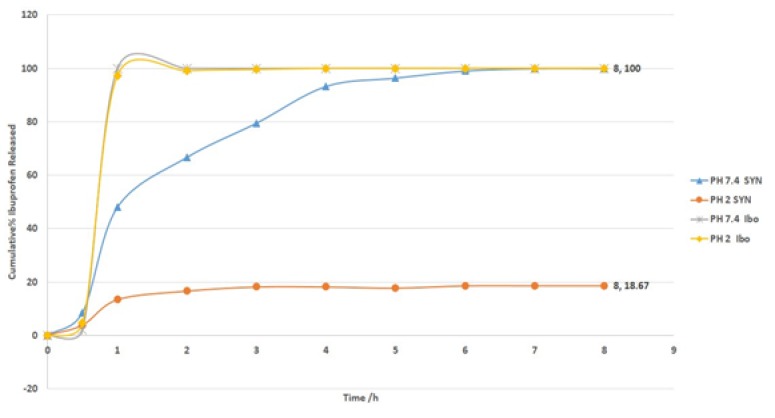
Cumulative percent of in vitro release of ibuprofen (SYN) from the microbeads and commercial ibuprofen at 37ºC in PBS, pH 7.4 and ibuprofen in HCl, pH 2.0

**In vitro study: **In vitro studies showed that ibuprofen is not released from the alginate microbeads when incubated in HCl (pH=2). However, when the beads were transferred to phosphate-buffered saline (PBS) at a pH of 7.4, all of the ibuprofen is released within 7h in a 1 active form (Figure 3). 

**Pharmacokinetics: **Pharmacokinetic indexes (T_max_, C_max_, and AUC_0–t_) are demonstrated in table 1 and figure1. The median Tmax of ibuprofen, ibuprofen arginine and solubilized ibuprofen capsule were 1.25, 0.42 and 0.5 hours, respectively. Ibuprofen has a significant difference with ibuprofen arginine and solubilized ibuprofen capsule (P<0.001). After the oral intake of 200 mg of ibuprofen, the C_max_ was lower in ibuprofen and the AUC_0–t_ was equivalent when evaluating the systemic exposures of ibuprofen and solubilized ibuprofen capsule compared to ibuprofen arginine. Also, the t½ and apparent clearance in the elimination process was not significantly different among the three groups (P=0.813 and P=0.906, respectively). 

The enteric-coated ibuprofen product tends to disintegrate longer than the ibuprofen tablet. Comparison of ibuprofen concentration shows a statistical difference between the two groups (table 1). Besides, a higher concentration was observed in the group receiving enteric-coated ibuprofen taking 4h after dosing. Ibuprofen concentration in serum increased rapidly to the maximum level after 1 hour in the group receiving regular tablet form and increased enteric-coated after 4 hours. The maximum and minimum concentrations of ibuprofen at 1 hour were 11.7 and 0.01 µg/ml for ibuprofen tablet and 10.32 and 0.82 µg/ml for enteric-coated form, respectively (table 1). 

**Table 1:** Pharmacokinetic parameters of ibuprofen after single oral administration of ibuprofen tablet (200 mg) and (ibuprofen rnteric-coated capsule (200 mg) in healthy volunteers

**Parameter**	**Ibuprofen tablet**	**Ibuprofen entric-coated capsule**
T max (hours)	1	4
Cmax (µg/ml)	11.71	10.32
AUC_0–t _(mg⋅h/l)	61.51	82.62*

Cmax: maximum concentration;

Tmax: time to Cmax;

AUC: area under the blood level curve

* Significant differences at p ≤ 0.05 between two groups

**Figure 4:**
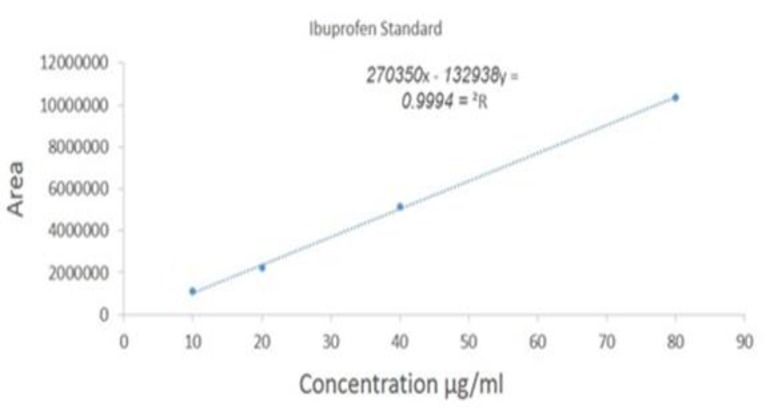
Linearity plot for ibuprofen standards at 10, 20, 40 and 80 µg/ml

**Figure 5:**
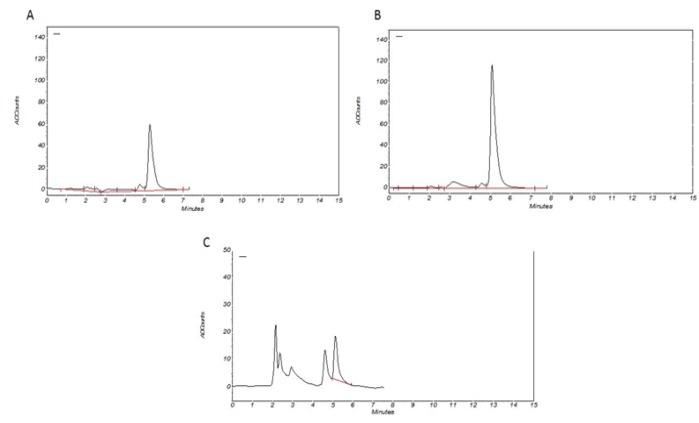
The sample HPLC peaks of ibuprofen (RT: 5.4 min). A) Standard ibuprofen (10µg/ml); B) standard ibuprofen (20µg/ml); C) serum sample after 1h ibuprofen received

**The standard curve** **: **To determine the concentration of the serum samples, the standard curve was plotted with a minimum of 4 concentrations of ibuprofen in the standard solution (figures 4, 5). Standard curves of ibuprofen at 10, 20, 40 and 80 µg/mL were prepared. R^2^ (linearity) parameter is also depicted in the chart. The coefficient of variation (CV) was obtained based on the area under the curve, which was 4.75%.

**Safety and tolerability: **There were no adverse events relevant to drugs of our research. There was no report about clinically significant physical checkup findings, vital signs, laboratory abnormalities and electrocardiogram results. None of these adverse events were discontinued in our research.

**Figure 6:**
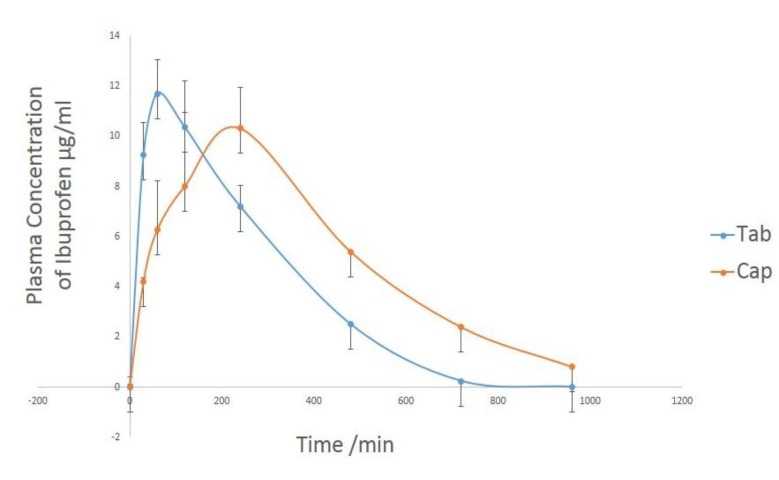
Mean and standard deviation of plasma concentration–time profiles of ibuprofen in all healthy subjects after a single 200 mg oral dose

## Discussion

The NSAIDs’ GI (gastro-intestinal) side effects have been widely reported, especially in patients with painful chronic diseases (20). To avoid the GI side effects, enteric-coated forms have been suggested (16). The results of this study have shown that the designed enteric-coated dosage form of ibuprofen can deliver ibuprofen in the gut in a controlled manner. However, benefitting from such a biogel-based delivery system requires more detailed studies, mostly focused on the gut tissue absorption mechanism. Enteric-coated drugs remain unaffected by the acidic medium of the stomach and deliver their active material to the alkaline setting of the gut. Additionally, enteric-coatings are used in many other cases, including in drugs that are sensitive to the gastric acid, e.g. pantoprazole, or in cases where the drug is required to affect the gut tissue directly, such as sulfasalazine (21). 

 To escape gastric degradation, this kind of dosage forms should not be crushed, chewed, or split in half when administered. It is also recommended to receive the dosage forms on an empty stomach, otherwise there is a possibility of the mentioned enteric-coated drugs to disintegrate in the medium. The oral bioavailability of a drug depends on the target of delivery, the pathway of drug absorption, the physiological conditions, and the physicochemical properties of the compounds. 

Ibuprofen, as a weak acid (pKa= 4.4), is able to dissolve in acidic pH media. This property could have the key effect on plasma drug concentration. To enhance water solubility, manufacturing salt forms of the acidic drugs have been suggested as a practical method, one that does not affect the drug bioavailability (22).

It has been previously reported that the more soluble form of ibuprofen shows a higher rate of absorption (lower T_max _ and higher C_max_) compared to the regular drug form (15, 23). 

Additionally, based on a clinical trial after oral administration of single dose (200 mg) of two formulations of ibuprofen, the C_max_ of arginine ibuprofen was shown to be equal to regular ibuprofen, while the average T_max_ for ibuprofen was significantly delayed compared to the other dosage form. As a result, formulating more soluble drug forms leads to a higher rate of absorption, followed by a larger C_max_ and smaller T_max_ which happens while preserving the other pharmacokinetic processes. In general, if the goal is to manage severe pains more rapidly and efficiently, formulations with a faster rate of absorption are suggested, where decreasing the drug concentration or delaying drug delivery might fail the treatment process. On the contrary, the use of such rapidly-absorbable formulations in patients with GI problems will exacerbate the pain and fail the treatment. 

To avoid GI damages in NSAID’s administration, in this study we managed to design a method for making enteric-coated ibuprofen. The serum samples were analyzed using the method of HPLC, each individual having received 200 mg of the prepared ibuprofen (and in a parallel study, the more soluble form). It was observed that the T_max_ of the prepared drug is 4 hours after drug administration, which is delayed 2 hours compared with the soluble ibuprofen. Additionally, the AUC of the enteric-coated was reported (figure 6) to be higher than that of the soluble ibuprofen. 

In conclusion, the obtained results from both in vivo and in vitro trials show that our formulation of the enteric coated ibuprofen can bypass the acidic environment of the stomach as well as delivering in the small intestine. Such administration of ibuprofen seems to be more useful for patients with gastrointestinal diseases. Additionally, in the mentioned property, this formulation can maintain an adequate level of drug concentration in the blood serum at least 4 hours after administration, which makes it a more effective painkiller through larger time intervals.
